# CWP232228 targets liver cancer stem cells through Wnt/β-catenin signaling: a novel therapeutic approach for liver cancer treatment

**DOI:** 10.18632/oncotarget.7954

**Published:** 2016-03-07

**Authors:** Ji-Young Kim, Hwa-Yong Lee, Kwan-Kyu Park, Yang-Kyu Choi, Jeong-Seok Nam, In-Sun Hong

**Affiliations:** ^1^ Center of Animal Care and Use, Lee Gil Ya Cancer and Diabetes Institute, Gachon University, Incheon, Republic of Korea; ^2^ Department of Laboratory Animal Medicine, College of Veterinary Medicine, Konkuk University, Seoul, Korea; ^3^ The Faculty of Liberal Arts, Jungwon University, Chungbuk, Republic of Korea; ^4^ Department of Pathology, College of Medicine, Catholic University of Daegu, Daegu, South Korea; ^5^ School of Life Sciences, Gwangju Institute of Science and Technology, Gwangju, Republic of Korea; ^6^ Laboratory of Stem Cell Research, Lee Gil Ya Cancer and Diabetes Institute, Gachon University, Incheon, South Korea; ^7^ Department of Molecular Medicine, School of Medicine, Gachon University, Incheon, Republic of Korea

**Keywords:** CWP232228, cancer stem cells, Wnt/β-catenin signaling, CD133, ALDH

## Abstract

Liver cancer stem cells (CSCs) are resistant to conventional chemotherapy and radiation, which may destroy tumor masses, but not all liver CSCs contribute to tumor initiation, metastasis, and relapse. In the present study, we showed that liver CSCs with elevated Wnt/β-catenin signaling possess much greater self-renewal and clonogenic potential. We further documented that the increased clonogenic potential of liver CSCs is highly associated with changes in Wnt/β-catenin signaling and that Wnt/β-catenin signaling activity is positively correlated with CD133 expression and aldehyde dehydrogenase (ALDH) enzymatic activity. Notably, the small molecule inhibitor CWP232228, which antagonizes the binding of β-catenin to TCF in the nucleus, inhibits Wnt/β-catenin signaling and depletes CD133^+^/ALDH^+^ liver CSCs, thus ultimately diminishing the self-renewal capacity of CSCs and decreasing tumorigenicity *in vitro* and *in vivo*. Taken together, our findings suggest that CWP232228 acts as a candidate therapeutic agent for liver cancer by preferentially targeting liver CSCs.

## INTRODUCTION

Hepatocellular carcinoma (HCC, also called malignant hepatoma), the most common type of liver cancer, is the fifth most frequently diagnosed solid tumor and the third leading cause of cancer death in the world [1, 2]. The molecular pathogenesis of liver cancer is not fully understood but is known to involve multiple genetic and/or epigenetic changes and the disruption of several important signaling pathways, including the Wnt/β-catenin [3], MAPK/ERK1/2 [4], PI3K/Akt [5], IGF-I [6], and VEGF [7] signaling pathways. Among these activated signaling pathways, Wnt/β-catenin signaling plays crucial roles in the development of HCC [8] and is generally regarded as one of the most difficult pathways to inhibit [9]. Importantly, aberrant Wnt/β-catenin signaling activation has been observed in at least 30% of human HCCs, and β-catenin mutations frequently occur in 15% to 30% of human HCCs [10].

Recently, it has been proposed that a small subset of cancer cells called cancer stem cells (CSCs) contribute to tumor initiation, growth, and invasion/metastasis [11]. CSCs have recently been identified in nearly all major cancer types, including leukemia [12] and breast [13], colon [14], and liver cancers [15]. Notably, liver CSCs are resistant to conventional anticancer therapies such as chemotherapy [16] and radiotherapy [17]. In this context, novel compounds and therapeutic strategies that focus on the selective targeting of liver CSCs will ultimately improve liver cancer patient outcomes and survival. Interestingly, accumulating evidence indicates that the activation of Wnt/β-catenin signaling is critical for the self-renewal of liver CSCs [18]. Wnt proteins are a large family of secreted cysteine-rich glycoproteins that play a critical role in regulating development of various organisms [19]. The dysfunction of the Wnt/β-catenin signaling pathway is associated with multiple types of human cancers, including breast [20], colon [21], and ovarian [22]. Indeed, liver CSCs with elevated Wnt/β-catenin signaling possess much greater self-renewal capacity and tumorigenicity than counterpart cells with low Wnt/β-catenin signaling activity [4, 23]. Thus, Wnt/β-catenin signaling is likely a promising therapeutic target for the treatment of liver cancer. However, few therapeutic agents specifically targeting Wnt/β-catenin signaling are currently available or under investigation [24].

The physical interaction between β-catenin and TCF is essential for the activation of Wnt/β-catenin signaling [25]. However, β-catenin has been traditionally considered as an ‘undruggable’ target because it possesses no discernible intrinsic enzymatic activity [26]. Recent studies have showed that several small synthetic compounds effectively inhibited oncogenic Wnt/β-catenin signaling by targeting β-catenin in various cancer types. Although recently synthesized small molecule compounds targeting β-catenin, such as IWP-2 [27] and XAV939 [28], successfully inhibited the interaction between β-catenin and TCF *in vitro*, the poor *in vivo* pharmacodynamics of these compounds have prevented their clinical application. Recently, our group demonstrated that CWP232228 (U.S. Patent 8,101,751 B2), a small molecule synthetic compound that antagonizes the binding of β-catenin to TCF in the nucleus, suppresses tumor formation and metastasis without toxicity through the inhibition of the growth of breast CSCs and bulk tumor cells *in vitro* and *in vivo* [29]. In the present study, we demonstrated for the first time that CWP232228 suppresses liver cancer formation by targeting liver CSCs through a molecular mechanism involving Wnt/β-catenin signaling. Taken together, these results suggest that using the small molecule β-catenin inhibitor CWP232228 to target liver CSCs, which are highly resistant to chemotherapy and are responsible for tumor relapse, may have significant clinical potential for the treatment of liver cancer.

## RESULTS

### Aberrant activation of Wnt/β-catenin signaling is associated with tumor progression in HCC

**Figure 1:**
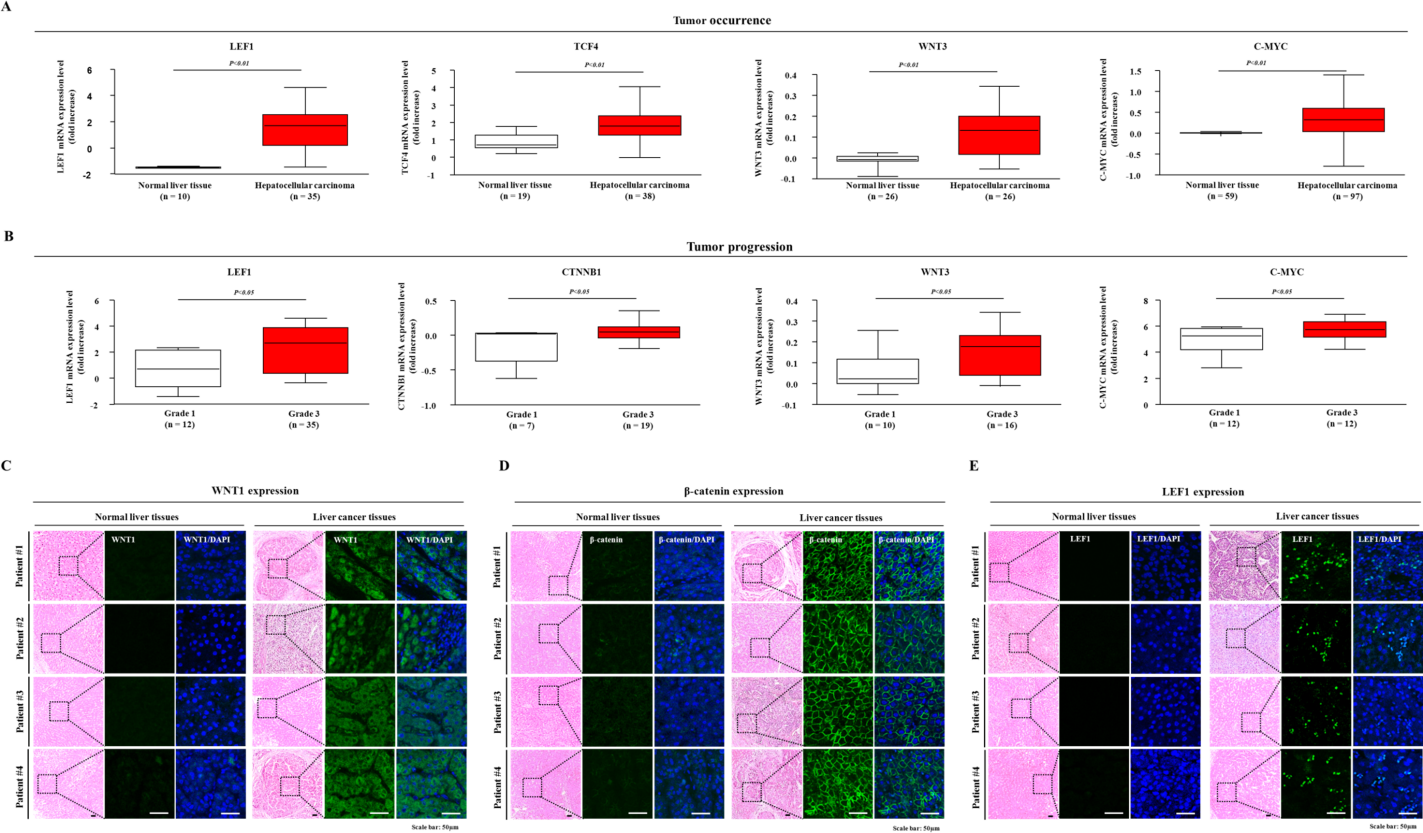
Expression profiles of Wnt/β-catenin signaling components in liver cancer patients A significant correlation between tumor occurrence and progression of hepatocellular carcinoma patients and the expression of Wnt/β-catenin signaling components was observed in Wurmbach dataset and Mas dataset, which were obtained through the Oncomine dataset repository (www.oncomine.org) (**A-B**). Normal and liver cancer tissues (kindly provided by Dr. Kwan-Kyu Park at the Catholic University, South Korea) were stained with antibodies against WNT1, β-catenin, and LEF1. These Wnt/β-catenin signaling components were expressed to a greater extent in the cancerous tissues than in the non-cancerous tissues. DAPI staining was performed to label the nuclei within each field (**C-E**). These results are shown that Wnt/β-catenin signaling may contribute to liver carcinogenesis. The results are presented as the mean ± SD.

Recent evidence has revealed the regulatory role of Wnt/β-catenin signaling in maintaining liver CSCs [18, 30]. Thus, to investigate the correlation between the expression patterns of Wnt/β-catenin signaling components and patient survival or liver cancer prognosis, we analyzed the available liver cancer data repositories in the Oncomine database (www.oncomine.org). We observed significant correlations between the expression of Wnt/β-catenin signaling components and the occurrence/progression of tumors (Figure 1A–1B). Interestingly, we also observed significant correlations between the enhanced expression of Wnt/β-catenin signaling components and poor response to chemotherapeutic reagents (Supplementary Figure 1). Based on our findings, we propose that Wnt/β-catenin signaling might play a critical role in the self-renewal and tumorigenic capacities of liver CSCs. Therefore, to determine whether Wnt/β-catenin signaling is implicated in hepatocarcinogenesis, we examined the expression of Wnt/β-catenin signaling components, including Wnt1, LEF, and TCF4, in tissue samples from liver cancer patients. As shown in Figure 1C–1E, we confirmed that Wnt1, LEF, and β-catenin-positive cell populations were significantly increased in human liver cancer tissues. These results suggest that Wnt/β-catenin signaling may contribute to tumorigenesis. Thus, the Wnt/β-catenin signaling pathway represents a potential therapeutic target for specifically eliminating liver CSCs.

### Wnt/β-catenin signaling-associated components are enriched in sphere-forming subpopulations

**Figure 2:**
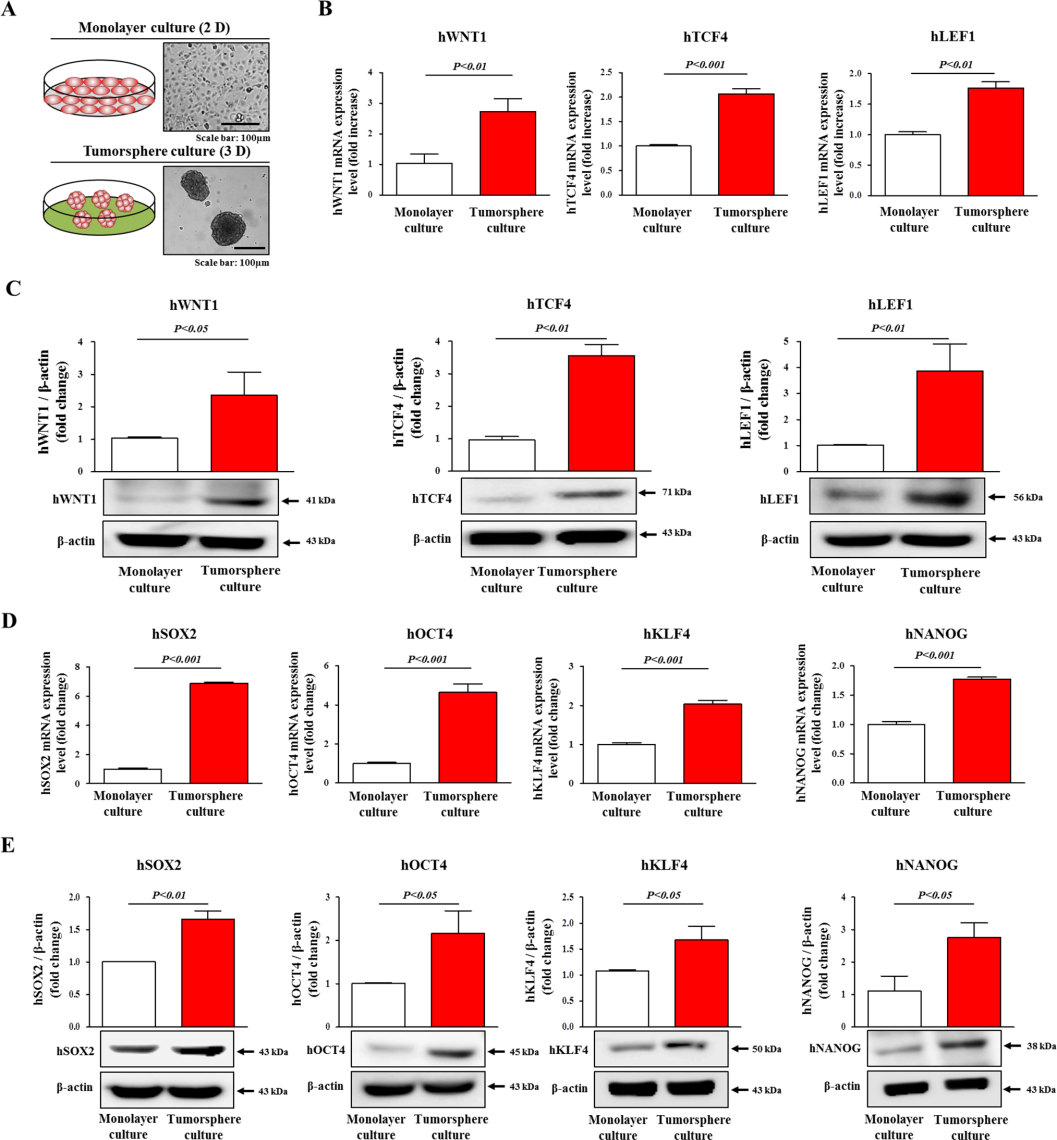
Sphere-forming Hep3B cell culture leads to expression Wnt/β-catenin signaling components and stem cell markers The mRNA and protein levels of WNT1, TCF4, and LEF1 in Hep3B monolayer and sphere-forming cells were measured using real-time PCR (**B**) and Western blotting (**C**). Real-time PCR (**D**) and Western blotting (**E**) results demonstrating changes in the expression of the stem cell markers SOX2, OCT4, KLF4, and NANOG after one week in sphere culture relative to Hep3B cells in sub-confluent monolayers. These results are suggested that our 3D culture system is suitable for evaluation of hepatic stem cells-like properties. β-actin was used as an internal control. The results are presented as the means ± SD from three independent experiments.

Previous studies have been suggested that stem/progenitor-like cell populations are enriched in sphere cell culture in multiple cancer types, including breast [31], colon [14], brain, and pancreatic [32] cancers. Therefore, to confirm whether sphere-forming culture is particularly useful for enriching the potential of liver CSCs, we examined the expression profiles of Wnt/β-catenin signaling components (Wnt1, LEF, and TCF4) under three-dimensional (3D) culture conditions. Consistent with our hypothesis, both the mRNA and protein levels of these components were higher in sphere-forming Hep3B cells than in cells in monolayers (Figure 2A–2C). In accordance with the results from Hep3B cells, the mRNA levels of these components were higher in sphere-forming Huh7 and HepG2 cells than in cells in monolayers (Supplementary Figure 2). Furthermore, recent studies have shown that the stem cell markers Oct4 [33], Sox2 [34], Nanog [35], and Klf4 [36] play important roles in regulating the self-renewal of liver CSCs. As expected, both the mRNA and protein levels of these markers were higher in sphere-forming Hep3B cells than in Hep3B cells in monolayers (Figure 2D–2E). Consistent with the results from Hep3B cells, the mRNA levels of these stemness-related markers were higher in sphere-forming Huh7 and HepG2 cells than in cells in monolayers (Supplementary Figure 3). These results indicate that our 3D culture conditions can be used to generate liver CSCs as an *in vitro* model to evaluate the efficacy of Wnt/β-catenin signaling inhibitors.

### CD133^+^/ALDH^+^ HCC cells possess increased *in vitro* clonogenic ability

**Figure 3:**
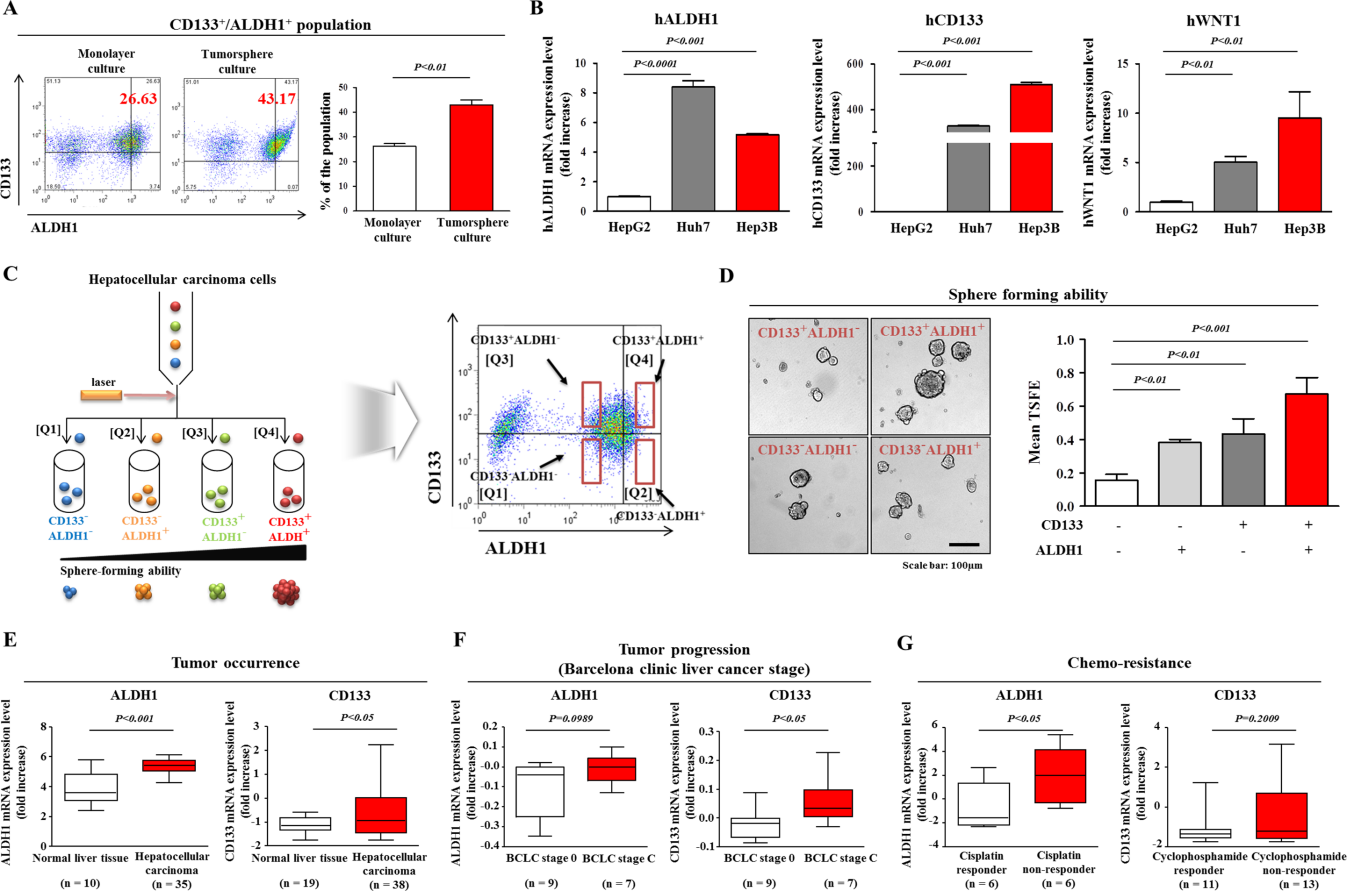
Constitutive expression of stem cell marker CD133 and ALDH1 is a hallmark of clonogenic potential and maintenance of liver CSCs The results of FACS analysis showed the increased percentage of CD133- and ALDH1-positive cell subpopulation in the total population of Hep3B cells in both monolayer and sphere cultures (**A**). The expression levels of WNT1, ALDH1 and CD133 were measured in various HCC cell lines (HepG2, Huh7, and Hep3B) using real-time PCR (**B**). Hep3B cells were sorted by dual-color flow cytometry analysis according to CD133 expression and ALDH1 activity. The dot plot is divided into four quadrants for CD133^+^ALDH1^+^, CD133^+^ALDH1^−^, CD133^−^ALDH1^+^ or CD133^−^ALDH1^−^(**C**). The sorted Hep3B cell populations were plated into sphere-forming culture dishes and their clonogenic abilities were analyzed (**D**). These results are indicated that CD133 and ALDH1 activity can lead to enhanced clonogenic ability. A significant correlation between tumor occurrence/prognosis or chemoresistance of liver cancer and the expression of CD133 and ALDH1 was observed in Gyoffy dataset, which were obtained through the Oncomine dataset repository (www.oncomine.org) (**E-G**). The results represent the means ± SD from three independent experiments.

Previous studies have demonstrated that liver CSCs can be recognized by multiple cell surface markers. For example, CD133 positive subpopulations obtained from HCC cells have a greater potential to develop tumors *in vivo* and exhibit hepatic stem/progenitor cell characteristics, including stem cell specific gene expression, self-renewal capacity, and multi-lineage differentiation potential [37]. In addition, a recent result also showed that aldehyde dehydrogenase (ALDH) is positively correlated with CD133^+^ HCC subpopulations [38]. These results suggest that the co-expression of ALDH with CD133 is more specific to the tumorigenic liver CSC subpopulation. We therefore performed fluorescence-activated cell sorting (FACS) analysis to quantitate the percentage of ALDH^+^ and CD133^+^ cells within a total cell population in both 2D monolayer and 3D sphere cultures. As expected, the percentage of double-positive Hep3B cells expressing these liver CSC markers was significantly higher in sphere-forming culture than in monolayer culture (Figure 3A). Next, to determine whether hepatocarcinogenesis is associated with changes in Wnt/β-catenin signaling or whether its signaling activity is positively correlated with CD133 expression and ALDH enzymatic activity, we compared the expression patterns of ALDH1, CD133, and WNT1 in several liver cancer cell lines, including HepG2, Huh7, and Hep3B cells, by real-time PCR analysis. Enhanced expression of the Wnt/β-catenin signaling component WNT1 was positively correlated with higher CD133 and ALDH levels; that is, those cell lines with higher WNT1 expression, such as Huh7 and Hep3B cells, correspondingly had higher CD133 and ALDH levels (Figure 3B). To further analyze the clonogenic potential of isolated Hep3B cells using those liver CSC markers, CD133^+^ALDH1^+^, CD133^+^ALDH1^−^, CD133^−^ALDH1^+^ or CD133^−^ALDH1^−^ cells were isolated by dual-color flow cytometry analysis (Figure 3C). Subsequent studies on purified subpopulations revealed the existence of a hierarchical clonogenic potential in the following order: CD133^+^ALDH1^+^, CD133^−^ALDH1^+^, CD133^+^ALDH1^−^, and CD133^−^ALDH1^−^ (Figure 3D). To further confirm the connection between hepatocarcinogenesis and CD133/ALDH1 expression, we analyzed the available liver cancer data repositories in the Oncomine database (www.oncomine.org). We observed significant correlations between poor prognosis/chemoresistance and high CD133 and ALDH1 expression (Figure 3E–3G). These results suggest that CD133 expression and ALDH1 enzymatic activity could contribute to the clonogenic potential of liver CSCs and might be associated with poor prognosis in liver cancer.

### The small molecule inhibitor CWP232228 effectively inhibits the clonogenicity of liver CSCs

**Figure 4:**
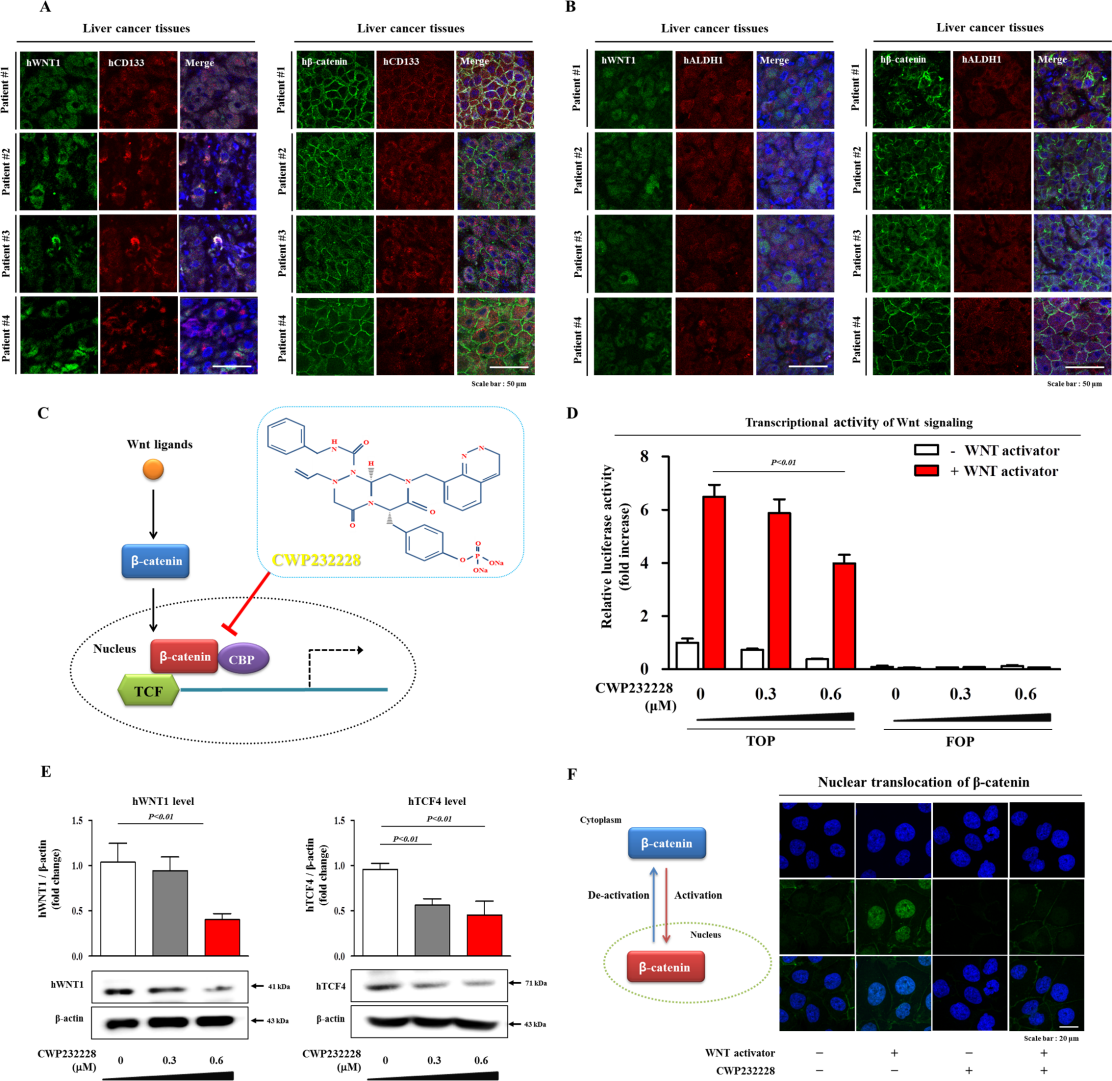
The effects of CWP232228 on Wnt/β-catenin signaling related components Normal and liver cancer tissues (kindly provided by Dr. Kwan-Kyu Park at the Catholic University, South Korea) were were co-stained with antibodies specific for CD133 and Wnt/β-catenin signaling components WNT1, LEF1, and β-catenin. Wnt/β-catenin signaling components-positive cells largely overlapped with ALDH1- and CD133-positive cells in the cancerous tissues (**A-B**). The chemical structure and molecular mechanism of CWP232228 (**C**). β-catenin responsive TOPFlash luciferase assays revealed that CWP232228 inhibits recombinant Wnt ligand-induced Wnt/β-catenin signaling in Hep3B cells. CWP232228 treatment strongly attenuated ligand-induced TOPFlash activity (**D**). The inhibitory effect of CWP232228 on the expressions of Wnt/β-catenin signaling target gene WNT1 and TCF4 were assessed in Hep3B cells through western blot analysis (**E**). The stimulatory effects of Wnt ligand on the nuclear translocation of β-catenin were successfully attenuated after CWP232228 treatment(**F**). Hep3B cells were stained using an antibody specific for β-catenin. These results are suggested that CWP232228 is significantly inhibited the activity of Wnt/β-catenin signaling components in Hep3B cells. Abbreviations: FOPFlash: a reporter plasmid containing mutant Tcf-binding sites, TOPFlash: a reporter plasmid containing multiple copies of wild-type Tcf-binding sites. Topflash and Fopflash (Addgene, Cambridg, MA, USA) reporter constructs is also known as pcDNA3.0 plasmids (Promoter: CMV; Cloning method: restriction enzyme; Size: 5428; Bacterial resistance: Ampicillin; 5′ sequencing 1 primer: CMV-F; 3′ sequencing 1 primer:BGH-rev.) DAPI staining was performed to label the nuclei within each field. β-actin was used as an internal control. The results represent the means ± SD from three independent experiments.

**Figure 5:**
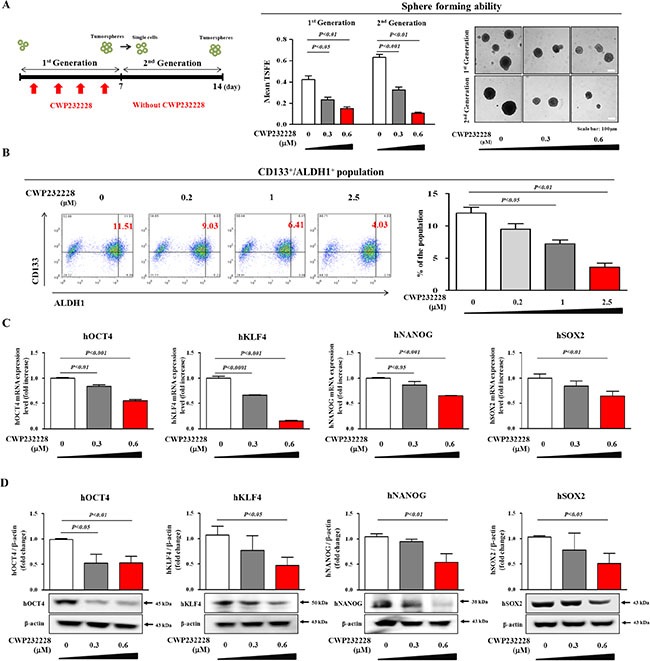
Effect of CWP232228 on the growth and clonogenicity of liver CSCs CWP232228 inhibited primary (with CWP232228 treatment) and second sphere formation (without additional CWP232228 treatment) in Hep3B cells. The sphere sizes greater than 100 μm were enumerated, and a representative image of a tumor sphere is shown (**A**). The percentage of ALDH1 and CD133 subpopulations were evaluated by FACS analysis. The treatment of Hep3B cells with CWP232228 for 48 h decreased the percentage of ALDH1 and CD133- double positive cells in the total cell population (**B**). The inhibitory effect of CWP232228 on the expression of stem cell markers OCT4, KLF4, NANOG, and SOX2 was assessed in Hep3B cells through real-time PCR (**C**) and western blot analysis (**D**). These results are indicated that CWP232228 is significantly decreased the mRNA and protein levels of hepatic stem cell markers. Abbreviations: TSFE, Tumor sphere-forming efficiency. β-actin was used as an internal control. The results represent the means ± SD from three independent experiments.

To investigate the correlations between Wnt/β-catenin signaling and ALDH/CD133-positive liver CSC populations in liver cancer patients, we analyzed the expression of major Wnt/β-catenin signaling-associated components (Wnt1 and β-catenin) in ALDH- and/or CD133-positive tissue samples from HCC patients. As shown in Figure 4A–4B, cells that were positive for Wnt/β-catenin signaling components Wnt1 and β-catenin were largely also positive for ALDH1 and CD133 in HCC tissue samples. These data indicate that Wnt/β-catenin signaling may play a role in hepatocarcinogenesis, revealing this pathway as a potential therapeutic target for specifically eliminating liver CSCs. Next, we evaluated the efficacy of the small molecule β-catenin inhibitor CWP232228 (Figure 4C) to inhibit Wnt/β-catenin signaling in Hep3B cells transfected with a luciferase reporter vector with or without Wnt/β-catenin activator treatment. Basal transcriptional activity of the Wnt/β-catenin luciferase reporter was significantly suppressed by CWP232228 treatment in a dose-dependent manner (Figure 4D). However, CWP232228 treatment slightly increased Annexin V positive apoptotic cells compared with non-treated cells, in a concentration dependent manner (Supplementary Figure 4). These results suggest that CWP232228-induced apoptosis may be at least partly affects the transcriptional activity of the Wnt/β-catenin luciferase reporter. We further examined whether CWP232228 treatment was sufficient to inhibit Wnt/β-catenin signaling in Hep3B cells. Interestingly, CWP232228 exposure significantly inhibited basal expression of the Wnt/β-catenin signaling-associated components WNT1 and TCF4, in a dose-dependent manner (Figure 4E). Consistent with the results described above, the WNT ligand treatment increased the nuclear localization and expression of β-catenin in the liver cancer cells and these effects were significantly attenuated by CWP232228 treatment (Figure 4F). An approximate 50% inhibitory concentration (IC_50_) was calculated using a dose-response curve (Supplementary Figure 5). We then examined the inhibitory effect of CWP232228 on both the primary and secondary CSC sphere formation of Hep3B cells. CWP232228 treatment resulted in disruption of the primary CSC sphere-forming capacity in a dose-dependent manner (Figure 5A). For the secondary CSC sphere formation assay, the Hep3B cells from primary CSC spheres were re-plated on culture dishes without additional CWP232228 treatment. Importantly, we found that the cells derived from CWP232228-treated primary spheres did not form subsequent secondary CSC spheres as efficiently as the cells derived from untreated primary CSC spheres (Figure 5A). We then hypothesized that CWP232228 might inhibit liver CSC sphere formation by suppressing levels of the CSC markers ALDH1 and CD133. Indeed, the percentage of ALDH1^+^ and CD133^+^ cells within the total Hep3B cells population was significantly decreased by CWP232228 treatment in a dose-dependent manner (Figure 5B). Furthermore, we also investigated the effects of CWP232228 treatment on the liver CSC sphere formation and the size of the ALDH1^+^/CD133^+^ subpopulation in other liable human hepatocellular carcinoma cell lines such as Huh7 and HepG2 cells. Approximate 50% inhibitory concentrations (IC50) of CWP232228 in these cell lines were calculated using a dose-response curve (Supplementary Figure 6). Consistent with the results from Hep3B cells, treatment with CWP232228 resulted in the disruption of sphere formation (Supplementary Figure 7) and ALDH1^+^/CD133^+^ subpopulation (Supplementary Figure 8) in both Huh7 and HepG2 cells. To further confirm the Wnt/β-catenin signaling-mediated effects on tumor sphere formation though alternative inhibition methods, we treated Hep3B cells with other well-known Wnt/β-catenin signaling inhibitors FH535 and IWR1. Approximate IC_50_ values were determined using a dose-response curve. In Hep3B cells, the IC_50_ values of these inhibitors were 1.233 μM and 14.51 μM, respectively (Supplementary Figure 9). Consistent with upper results, FH535 and IWR1 treatment significantly suppressed tumor sphere formation, in a dose-dependent manner (Supplementary Figure 10). Next, we examined the expression of stem cell markers, including OCT4, SOX2, NANOG, and KLF4, in both monolayer and sphere-forming Hep3B cells with or without CWP232228 treatment. Consistent with our hypothesis, the mRNA and protein levels of these stem cell markers were significantly decreased by CWP232228 treatment in both culture conditions (Figure 5C–5D and Supplementary Figure 11). Additionally, we conducted the additional sets of experiments to determine whether CWP232228-induced apoptotic effects may affect the inhibitory effects of CWP232228 on the clonogenic potential of liver CSCs. Interestingly, CWP232228 treatment slightly increased Annexin V positive apoptotic cells compared with non-treated cells, in a concentration dependent manner (Supplementary Figure 5). These results suggest that CWP232228-induced apoptosis may be at least partly involved in the inhibitory effects of CWP232228 on the clonogenic potential of liver CSCs.

### CWP232228 effectively inhibits hepatocarcinogenesis in a mouse xenograft model

**Figure 6:**
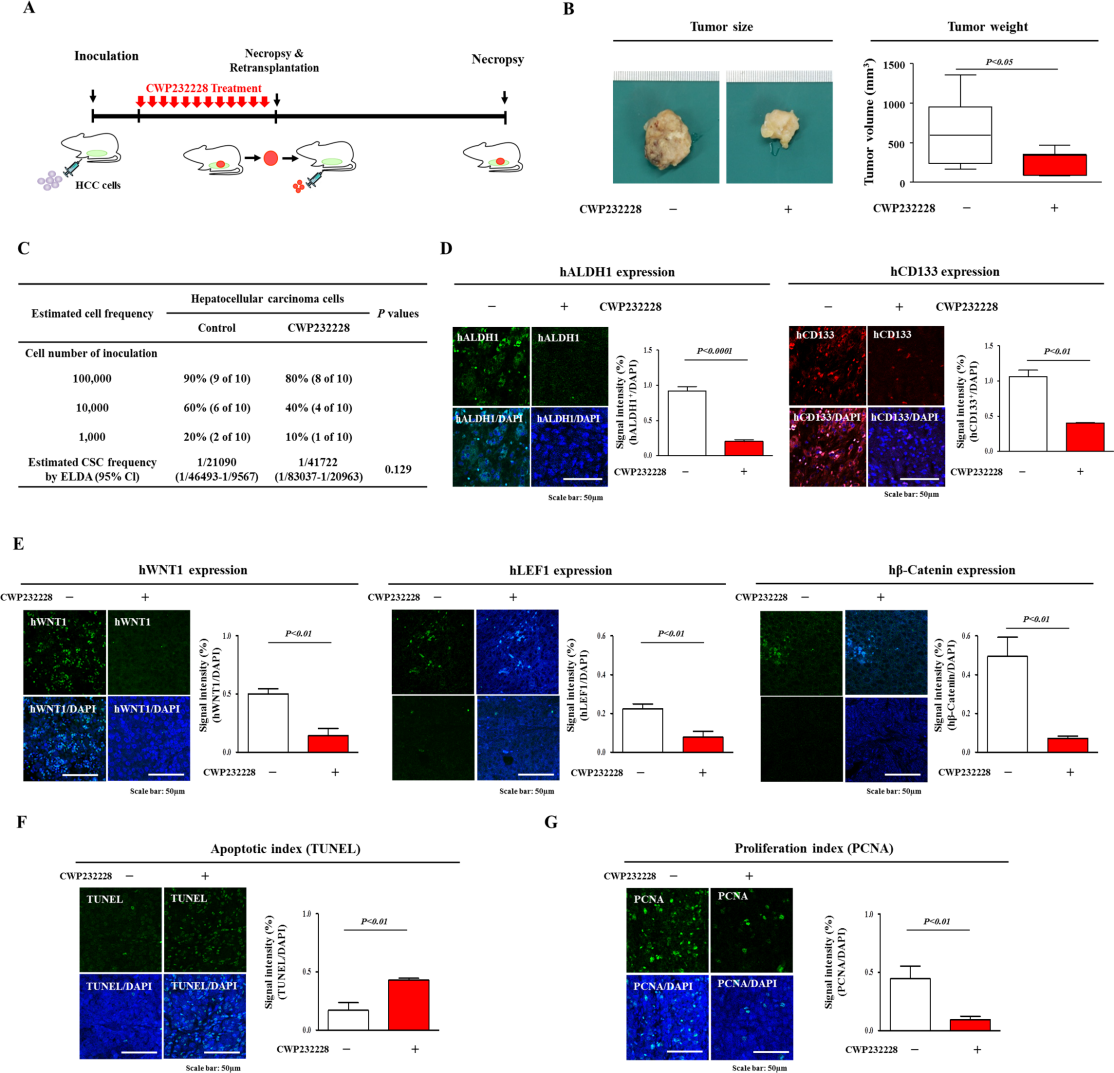
The effects of CWP232228 on hepatocarcinogenesis in a mice xenograft model Schematic representation of the experimental protocol as described in the materials and methods section (**A**). Anesthetized 7-weeks old male NOD/SCID mice were inoculated with 1:1 mix of matrigel and 5 × 10^5^ Hep3B cells into the subcutaneous tissue. Tumor tissue was isolated from mice bearing Hep3B cell tumors that had been treated with CWP232228 (100 mg/kg, intraperitoneally) or vehicle (PBS). Tumor volumes were measured as described in the materials and methods section (**B**). Hep3B cells from monolayer cultures or sphere cultures were dissociated into single cell suspensions and injected into the subcutaneous tissue of mice in limiting dilutions (1,000; 10,000; 100,000). Tumor formation was observed for 4weeks following inoculation. Liver CSC frequency was calculated using extreme limiting dilution assay (ELDA) (**C**). The ALDH1- and CD133-positive subpopulation, as a proportion of the total cell population in the tumor xenografts, was assessed by immunohistochemistry (**D**). The relative expression of Wnt/β-catenin signaling component WNT1, LEF1, and β-catenin in the tumor xenografts was assessed by immunohistochemistry (**E**). CWP232228-mediated apoptotic DNA fragmentation in tumor xenografts was visualized by TUNEL assay (**F**). Inhibitory effects of CWP232228 on the hepatocarcinogenesis were further confirmed by performing proliferating cell nuclear antigen (PCNA) immunohistochemistry to assess tumor xenografts (**G**). *In vivo* results are suggested that CWP232228 is inhibited tumor progression and decreased the expression levels of hepatic cancer stem markers and Wnt/β-catenin signaling components. DAPI staining was carried out to label the nuclei within each field. The results are presented as the mean ± SD from three independent experiments.

We further examined the *in vivo* effect of CWP232228 on Hep3B cells tumorigenesis. Importantly, CWP232228 treatment (100 mg/kg, intraperitoneal administration) resulted in a significant decrease in the size and weight of tumors compared with those of the control group (Figure 6A–6B). No obvious clinical symptoms, including anorexia, anuria, diarrhea, fecal changes, polyuria, excess salivation, and vomiting, were observed. We further evaluated the inhibitory effect of CWP232228 on hepatocarcinogenesis using extreme limiting dilution analysis (ELDA). The frequency of the repopulating unit was 1/21090 for the untreated control group and 1/41722 for CWP232228-treated group (Figure 6C). Therefore, CWP232228 reduced the repopulation frequency of tumor-initiating CSCs in xenograft mice. Then, to determine whether CWP232228 treatment affects Wnt/β-catenin signaling *in vivo*, we investigated the expression patterns of its signaling components, Wnt1, LEF1, and β-catenin in tumor tissues of mice with or without CWP232228 treatment. Consistent with our previous results, CWP232228 treatment led to a significant decrease in ALDH1/CD133-positive (Figure 6D) and Wnt/β-catenin-positive cells (Figure 6E). The inhibitory effects of CWP232228 on hepatocarcinogenesis *in vivo* were further confirmed by a terminal deoxynucleotidyl transferase dUTP nick end labeling (TUNEL) assay (Figure 6F) and by proliferating cell nuclear antigen (PCNA) staining (Figure 6G) of xenograft tumors.

## DISCUSSION

Liver cancer is the fifth most frequently diagnosed cancer and the second leading cause of cancer-related death among men in the world [39]. HCC represents the predominant histological subtype of primary liver malignancies, accounting for 70%–85% of the total liver cancer patients [39]. Despite numerous efforts to determine the cellular origin of HCC, whether HCC originates from mature hepatocytes or stem/progenitor cells remains unclear. Previous results have demonstrated that primary HCC is histologically and genetically heterogeneous and contains multiple cell types that preferentially express a number of cell surface markers [37, 40, 41]. A small subset of cancer cells called CSCs may contribute to tumor initiation, growth, invasion/metastasis, and recurrence [11]. To date, CSCs have been identified in nearly all major solid tumors, including breast [13], colon [14], and liver cancer [15]. Indeed, recent studies have suggested that the presence of functional liver CSCs in tumor samples may be associated with poor prognosis and an increased risk of metastasis in HCC patients who underwent radical dissection [42, 43]. Accordingly, CD133^+^ HCC cells were shown to have increased Akt signaling activity and resistance to chemotherapy drugs such as doxorubicin or 5-fluorouracil (FU), while an Akt inhibitor abolished the preferential survival of CD133^+^ HCC cells [44]. Therefore, therapeutic strategies that selectively target liver CSCs might ultimately improve liver cancer treatments.

Wnt/β-catenin signaling activation has been observed in at least 30% of all HCC patients. Mutations in β-catenin (a critical Wnt/β-catenin signaling component) occur in approximately 20% of HCC cases [45]. Indeed, the enhanced activation of β-catenin/TCF transcriptional activity has been associated with accelerated HCC tumorigenesis in a number of recent studies [46–48], suggesting that targeting the direct interaction of β-catenin with TCF rather than targeting other Wnt/β-catenin signaling regulators might effectively target liver CSCs. Despite the intensive investigation *in vitro* as well as *in vivo*, there is currently no FDA-approved small molecule Wnt/β-catenin signaling inhibitor for human use. The most of these inhibitors developed so far have focused on the preclinical or early clinical trials stage: JW55 (Tocris Bioscience) and XAV939 (Novartis Pharma) are in preclinical trials, and LGK974 (Novartis Pharma), OMP-18R5 (OncoMed Pharma/Bayer), PRI-724 (Prism Pharma) are in clinical phase I/II [29]. In this study, we used CWP232228 as a selective small molecule Wnt/β-catenin signaling inhibitor to antagonize the binding of β-catenin to TCF and thus to specifically suppress the expression of Wnt/β-catenin-responsive genes. Our group has previously used this compound to inhibit β-catenin-mediated transcription in murine and human breast cancer cells [29]. According to our previous study, a pharmacokinetic/pharmacodynamic (PK/PD) analysis demonstrated that CWP232228 administration in mice generated an exposure of this inhibitor in the blood at a concentration greater than 1 nmol/L for 7 h [29]. Moreover, No significant changes in mortality, body weight, hematologic values, and hemolytic potential were observed in our previous study [29], indicating that CWP232228-associated toxicity was minimal. The CWP232228-mediated inhibition of liver CSC clonogenicity (Figure 5A) and CSC marker expression (Figure 5C–5D) was illustrated by its ability to suppress β-catenin-mediated transcriptional activity (Figure 4D–4E). To the best of our knowledge, this is the first study to assess the ability of CWP232228 to inhibit liver cancer formation by targeting liver CSCs through the suppression of Wnt/β-catenin signaling. Further studies are required to investigate how the CWP232228-mediated inhibition of Wnt/β-catenin signaling influences the self-renewal properties of liver CSCs.

CD133 (also known as prominin-1, a surface membrane glycoprotein) is an important CSC surface marker in HCC [49, 50]. CD133^+^ HCC cells have previously been shown to have increased tumorigenicity and to be resistant to chemotherapy drugs via the activation Akt signaling and pro-survival Bcl-2 pathways [44]. In CD133^+^ HCC CSCs, the inactivation of Akt signaling by a specific inhibitor significantly reduced the levels of pro-survival Bcl-2 family proteins [44]. In subsequent studies, a CD133 antibody (AC133) conjugated to a chemotherapeutic agent effectively inhibited the growth rate of HCC cells *in vitro* and suppressed tumor growth in a xenograft model [51]. Collectively, these results suggest that targeting liver CSCs via CD133 might be a promising therapeutic strategy for liver cancer. Consistent with these results, FACS and Western blotting results also showed that CWP232228 treatment effectively disrupted the clonogenicity of the CD133^+^ CSC population (Figure 5B and Figure 6D). ALDH is a detoxifying enzyme that is involved in the oxidation of numerous aldehydes. Previous studies have suggested that increased ALDH activity is detected in human hematopoietic stem cells and that this increased activity promotes the self-renewal of these cells [52, 53]. More importantly, the potential role of ALDH in CSC chemoresistance has recently been revealed [54]. ALDH acts to detoxify various harmful intracellular aldehydes that would otherwise negatively affect healthy pluripotent stem or progenitor cell populations. Consistent with this result, recent studies have revealed that ALDH was differentially activated in the CD133^+^ subpopulation of various HCC cell lines [38]. Interestingly, not all CD133-expressing cells were ALDH positive, suggesting that ALDH, expressed in combination with CD133, further specifically characterizes the tumorigenic liver CSC subpopulation [38]. In this context, it seems reasonable and logical to speculate that targeting liver CSCs via ALDH might represent a novel therapeutic strategy for liver tumors. Indeed, subsequent studies in the present work revealed that the CWP232228-mediated suppression of Wnt/β-catenin signaling significantly decreased the number of ALDH^+^ cells (Figure 5B and Figure 6D).

In this study, to suppress the self-renewal and tumor-initiating capacities of liver CSCs through the inhibition of β-catenin-mediated Wnt signaling, we used CWP232228, a potent small molecule inhibitor that antagonizes the binding of β-catenin to TCF [29]. In response to CWP232228 treatment, the transcriptional activity of a Wnt/β-catenin luciferase reporter (Figure 4D–4E) and expression of the Wnt/β-catenin target genes WNT1, TCF4, and β-catenin (Figure 4F–4H) were significantly decreased in a dose-dependent manner. Similarly, CWP232228 treatment was sufficient to block primary and subsequent secondary liver CSC sphere formation *in vitro* (Figure 5A) and tumor development in the xenograft model (Figure 6A–6B). Moreover, the repopulating unit frequency of the basal population was also significantly reduced in the xenograft model (Figure 6C). These results suggest that CWP232228 inhibits the capacity of liver CSCs to initiate tumors. The marked reduction of ALDH- and CD133-positive liver CSC subpopulations by CWP232228 treatment provides further support of this interpretation (Figure 6D). In summary, our results provide clear evidence showing that the co-expression of ALDH and CD133 more specifically characterizes putative liver CSCs. Our data also show for the first time that the small molecule inhibitor CWP232228 inhibits Wnt/β-catenin signaling and depletes liver CSCs and thus will ultimately diminish the self-renewal capacity of CSCs and decrease their tumorigenicity. Further research should assess the effects of combination therapy using CWP232228 with other therapeutic drugs for liver cancer and the possibility of cross-talk between Wnt/β-catenin signaling and other signaling pathways.

## MATERIALS AND METHODS

### Cell culture and reagents

Hepatocellular carcinoma cell lines HepG2, Huh7, and Hep3B were obtained from the Korean cell line bank (Seoul, South Korea) and were cultured in DMEM (Invitrogen, Grand Island, NY) supplemented with 10% fetal bovine serum (FBS), 100 U/ml penicillin and 100 U/ml streptomycin (Lonza, Basel, Switzerland) at 37°C and 5% CO_2_. Wnt signaling inhibitor CWP232228 is designed by JW Pharmaceutical Corporation (Seoul, Korea).

### Tumorsphere formation

Single cells were resuspended in serum-free DMEM (Invitrogen) containing B27 (Invitrogen), 20 ng/ml EGF, 20 ng/ml bFGF (PeproTech) and 4 μg/ml heparin (Sigma-Aldrich). Primary tumorspheres were derived by plating 20,000 single cells/well into six-well ultra-low attachment dishes (Corning). Individual spheres ≥ 150 μm from each replicate well (*n* ≥ 9 wells) were counted under an inverted microscope at 50× magnification using the Image-Pro Plus program (Media Cybernetics). The percentage of cells capable of forming spheres, termed the ‘tumorsphere formation efficiency (TSFE)', was calculated as follows: [(number of sphere formed/number of single cells plated) × 100].

### Cell proliferation assay

Hep3B cells were seeded in 96-well plates. After 48 h of incubation, cell viability was assessed by cell counting kit-8 (Dojindo) according to the manufacturer's instruction. The numbers of viable cells were measured at a wavelength of 450 nm using Versamax microplate reader.

### Luciferase reporter assay

Hep3B cells were plated at a density of 2 × 10^4^ cells/well in 12-well plates, and transfected using Genefectine transfection reagent (Genetrone Biotech Co., Korea) according to the manufacturer's protocol. The TopFlash (Addgene, Cambridge, MA) [55], luciferase reporter (100 ng), and Renilla luciferase thymidine kinase construct (Invitrogen) (50 ng) were used to determine luciferase activity. Luciferase activity was measured by a luminometer (Glomax, Promega, Sunnyvale, CA), using a Dual-Luciferase assay kit (Promega), according to the manufacturer's recommendations. Total value of reporter activity in each sample was normalized to Renilla luciferase activity.

### Real-time PCR

Total RNA was extracted using TRIzol reagent (Invitrogen). RNA purity was verified by measuring 260/280 absorbance ratio. The first strand of cDNA was synthesized with 1 μg of total RNA using SuperScript II (Invitrogen), and one-tenth of the cDNA was used for each PCR mixture containing Express SYBR-Green qPCR Supermix (BioPrince, Seoul, Korea). Real-time PCR was performed using a Rotor-Gene Q (Qiagen). The reaction was subjected to 40-cycle amplification at 95°C for 20 sec, at 60°C for 20 sec and at 72°C for 25 sec. Relative mRNA expression of selected genes was normalized to PPIA and quantified using the DDCT method. The sequences of the PCR primers are listed in Table 1.

**Table 1:** Primer sequences quantitative RT-PCR

Gene	Genebank No.		Primer sequence
PPIA	NM_021130	F	TGCCATCGCCAAGGAGTAG
		R	TGCACAGACGGTCACTCAAA
WNT1	NM_005430	F	CGGGCAACAACCAAAGTC
		R	CAGCAGCAGCCTAGCAGAA
TCF4	NM_003199	F	CACGCCGGGAAACCCACCTC
		R	TGTCCTACGGTGCCAGGCGA
LEF1	NM_016269	F	AATAAAGTCCCGTGGTGC
		R	ATGGGTAGGGTTGCCTGAAT
SOX2	NM_003106	F	AAATGGGAGGGGTGCAAAAG
		R	CAGCTGTCATTTGCTGTGGG
KLF4	NM_004235	F	GAACTGACCAGGCACTACCG
		R	TTCTGGCAGTGTGGGTCATA
OCT4	NM_002701	F	ACATCAAAGCTCTGCAGAAAGAACT
		R	CTGAATACCTTCCCAAATAGAACCC
NANOG	NM_024865	F	ACATGCAACCTGAAGACGTGTG
		R	CATGGAAACCAGAACACGTGG
ALDH1	NM_000689	F	CCCCAGGAGTCACTCAAGGC
		R	CCCACGGGCCTCCTCCACAT
CD133	NM_006017	F	CAGAGTACAACGCCAAACCA
		R	AAATCACGATGAGGGTCAGC

### Flow cytometry

FACS analysis and cell sorting were performed using FACS Calibur and FACS Aria machines (Becton Dickinson, Palo Alto, CA), respectively. FACS data were analyzed using Flowjo software (Tree Star, Ashland, OR). Antibodies to the following proteins were used: PE-conjugated CD133 (dilution 1/40, MACS; Miltenyi Biotech, Sunnyvale, CA, Germany, 130-080-801) and PE-conjugated ALDH1 (dilution 1/40, StemCell Technologies, Durham, NC, USA, 01700). The FACS gates were established by staining with isotype antibody or secondary antibody.

### Protein isolation and western blot analysis

Protein expression levels were determined by western blot analysis as previously described. [56] Briefly, cells were lysed in a buffer containing 50 mM Tris, 5 mM EDTA, 150 mM NaCl, 1 mM DTT, 0.01% NP 40, 0.2 mM PMSF. The protein concentrations of the total cell lysates were measured by using bovine serum albumin as standard. Samples containing equal amounts of protein were separated by sodium dodecyl sulfate polyacrylamide gel electrophoresis (SDS-PAGE) and then transferred onto polyvinylidene difluoride (PVDF) membranes (Bio-RAD Laboratories). The membranes were blocked with 5% skim milk in Tris buffered saline containing Tween-20 at RT, and the membranes were with primary anti-β-actin (Abcam, Cambridge, MA, USA, ab189073), Oct4 (Abcam, Cambridge, MA, USA, ab19857), Klf4 (Abcam, Cambridge, MA, USA, ab72543), Nanog (Abcam, Cambridge, MA, USA, ab21603), Sox2 (Abcam, Cambridge, MA, USA, ab184149), Wnt1 (Abcam, Cambridge, MA, USA, ab15251), TCF4 (Abcam, Cambridge, MA, USA, ab72586), and LEF1 (Cell Signaling Technology, Beverly, MA, USA, 2230S) antibodies overnight at 4°C and then with HRP-conjugated goat anti-rabbit IgG (BD Pharmingen, San Diego, CA, USA, 554021) and HRP goat anti-mouse IgG (BD Pharmingen, San Diego, CA, USA, 554002) secondary antibodies for 90 min at RT. Antibody-bound proteins were detected using an ECL.

### Immunofluorescent staining

The use of fresh liver tumor specimens was approved by the research ethic committees at the Catholic University (South Korea). Informed consent was obtained from all patients. Samples were fixed with 4% paraformaldehyde for fluorescent staining. Samples were permeabilized with 0.4 M glycine and 0.3% Triton X-100, and nonspecific binding was blocked with 2% normal swine serum (DAKO, Glostrup, Denmark). Staining was performed as described previously [57], using the primary anti-ALDH1 (Abcam, Cambridge, MA, USA, ab52492), CD133 (Biorbyt, Cambridge, UK, orb114000), Wnt1 (Abcam, Cambridge, MA, USA, ab15251), β-catenin (Cell Signaling Technology, Beverly, MA, USA, #9562), and PCNA (Vector Laboratories, Burlingame, CA, USA, VP-RM04) antibody. Samples were examined by fluorescence microscopy (Zeiss LSM 510 Meta). The calculation of expression was based on green fluorescence area and density divided by cell number, as determined from the number of DAPI-stained nuclei, in three randomly selected fields for each sample from a total of three independent experiments.

### Tumorigenesis experiment

All animal experiments were carried out with the approval of IACUC (Institutional Animal Care and Use Committee) guidelines (No.LCDI-2012-0069). For tumorigenesis experiments, anesthetized 7-weeks old male NOD/SCID mice (Orient Charles River Technology, Korea) were inoculated with 1:1 mix of matrigel and 5 × 10^5^ Hep3B cells into the subcutaneous tissue. After inoculation, the mice were randomly assigned to knockdown groups and control group. And it was monitored for 8 weeks.

### *In vivo* limiting dilution assay

For the limiting dilution experiment, primary tumors were minced using scissors and incubated in DMEM (Invitrogen) mixed with collagenase/hyaluronidase (Stem cell Technologies) at 37°C for 15–20 min. Primary tumor-derived cells were inoculated into the subcutaneous tissue of mice at varying cell densities ranging from 1 × 10^3^ to 1 × 10^5^ cells. Hep3B cells-injected mice were euthanized on eight weeks. The frequency of tumor-initiating cells (TICs) was calculated using ELDA web tool (http://bioinf.wehi.edu.au/software/elda). The volume of the primary tumor was measured as previously described [33].

### TUNEL assay

Apoptotic cells in tumor section were detected by TUNEL system kit (Promega, Madison, WI, USA, G3250) according to the manufacture's protocol. Briefly, tumor tissues were fixed with 10% neutral buffered formalin. Tumor sections were removed paraffin, rehydrated, and permeabilized by proteinase K (20 μg/ml). Apoptotic cells were labeled with terminal deoxinucleotidyl transferase (TdT) solution for 1 hour at 37°C in a humidified chamber. All nuclei were counterstained with DAPI.

### Oncomine database analysis

We used Oncomine Cancer Microarray database (http://www.oncomine.org/) to analyze the expression levels of CSCs markers (CD133 and ALDH1) and Wnt/β-catenin signaling-related genes (LEF1, TCF4, WNT3, C-MYC and β-Catenin) in normal hepatic tissues and hepatocellular carcinoma tissues. These genes expression data were log2 transformed and median centered. All graphics and statistic values were analyzed by GraphPad Prism 5.0 and *p*-values calculated by two-tailed Student's *t*-test (*P* < 0.05).

### Statistical analysis

All the statistical data were analyzed by GraphPad Prism 5.0 (GraphPad Software, San Diego, CA) and evaluated by two-tailed Student's *t*-test. Value of *P* < 0.05 was considered to indicate statistical significance.

## SUPPLEMENTARY MATERIALS FIGURES


