# Supplementary material for: CWP232228 targets liver cancer stem cells through Wnt/β-catenin signaling: a novel therapeutic approach for liver cancer treatment
Source: Oncotarget. 2016 Mar 7;7(15):20395–409. doi: 10.18632/oncotarget.7954 (PMC4991463; doi:10.18632/oncotarget.7954)
Supplement: Supplementary file 1 [file oncotarget-07-20395-s001.pdf]

CWP232228 targets liver cancer stem cells through Wnt/β-catenin signaling: a novel therapeutic approach for liver cancer treatment

Supplementary Materials

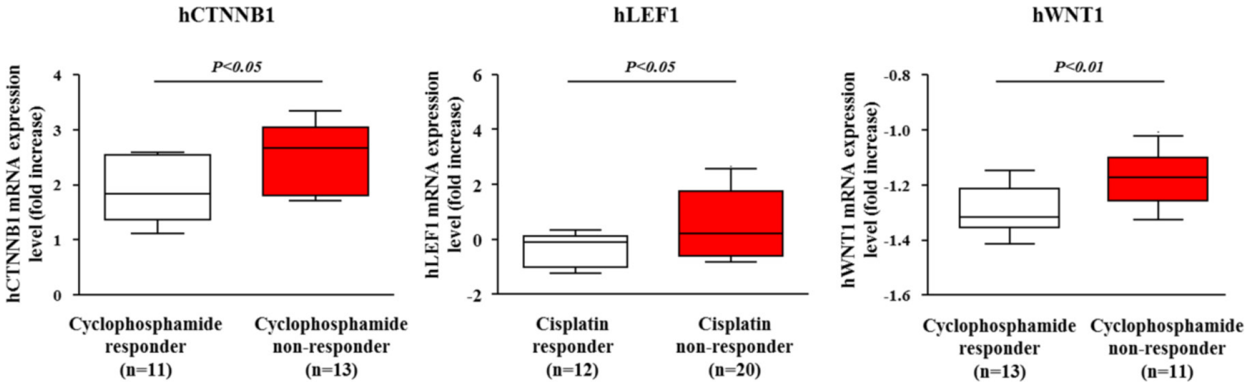

Supplementary Figure S1: The relative expression profiles of Wnt/β-catenin signaling components between responders and non-responders to chemotherapeutic agent. A significant correlation between response to chemotherapeutic agents and the expression of Wnt/β-catenin signaling components was observed in Gyorffy dataset and Wurmbach dataset, which were obtained through the Oncomine dataset repository (www.oncomine.org).

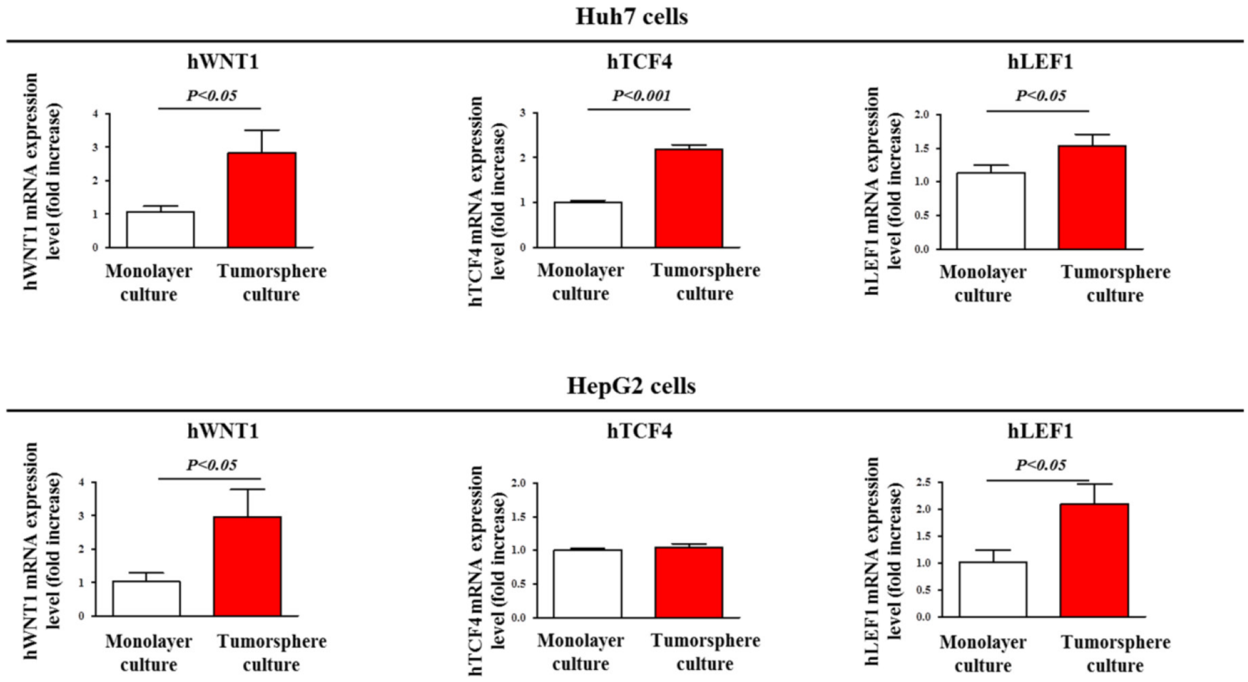

Supplementary Figure S2: Sphere-forming 3D culture condition leads to expression of Wnt/β-catenin signaling components. The mRNA levels of WNT1, TCF4, and LEF1 in monolayer and 3D sphere-forming cells were measured using real-time PCR. Wnt/β-catenin signaling components in sphere-forming cell subpopulations are significantly higher than that in the monolayer. The results are presented as the mean ± SD from three independent experiments.

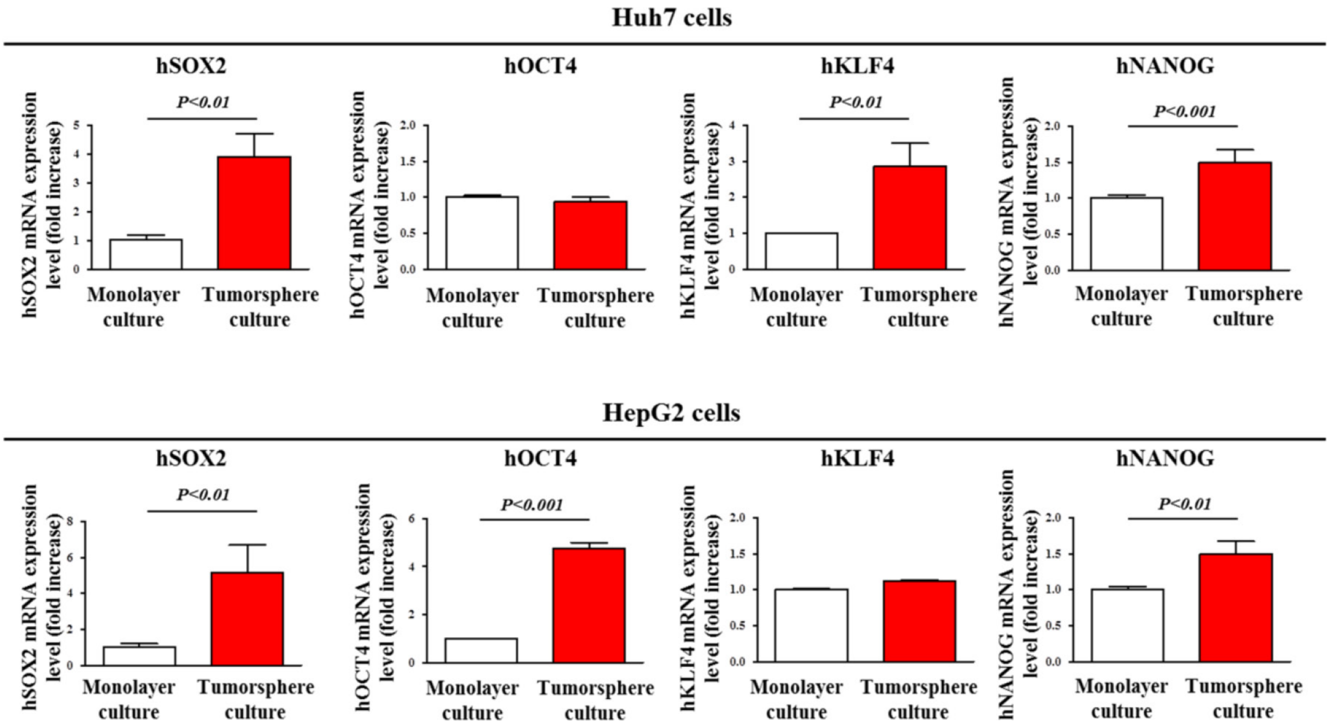

Supplementary Figure S3: Sphere-forming 3D culture condition leads to expression of stemness-related genes. The mRNA levels of SOX2, OCT4, KLF4, and NANOG in monolayer and sphere-forming cells were measured using real-time PCR. Stemness-related genes in sphere-forming cell subpopulations are significantly higher than that in the monolayer. The results are presented as the mean ± SD from three independent experiments.

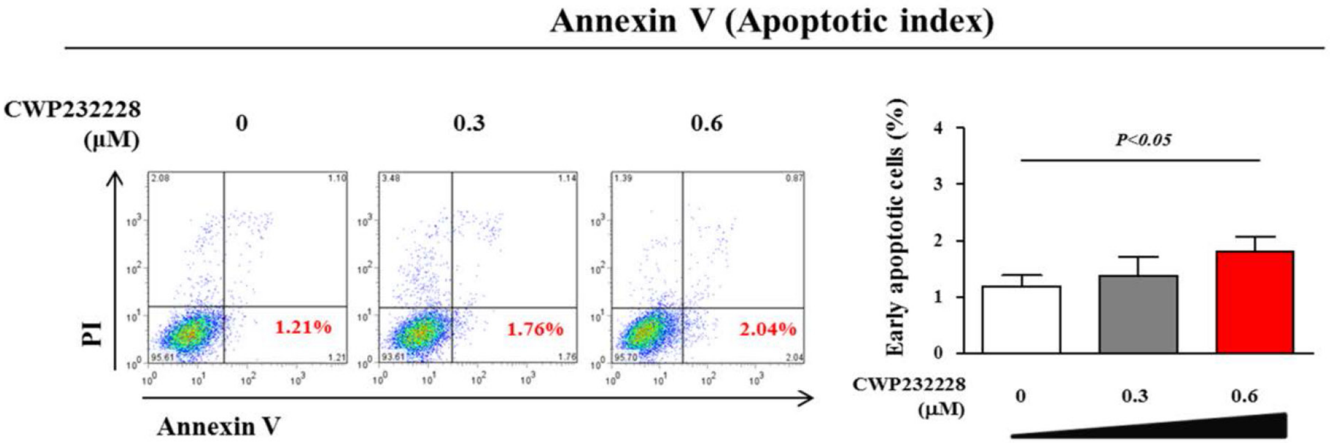

Supplementary Figure S4: The effects of CWP232228 on cell apoptosis in Hep3B cells. Cytotoxicity after CWP232228 treatment for 48 hours was determined using Annexin V-FITC binding assay in Hep3B cells. The percentage of early apoptotic cells (Annexin V+, PI– quadrant) were calculated and shown in histogram. The results are presented as the mean ± SD from three independent experiments.

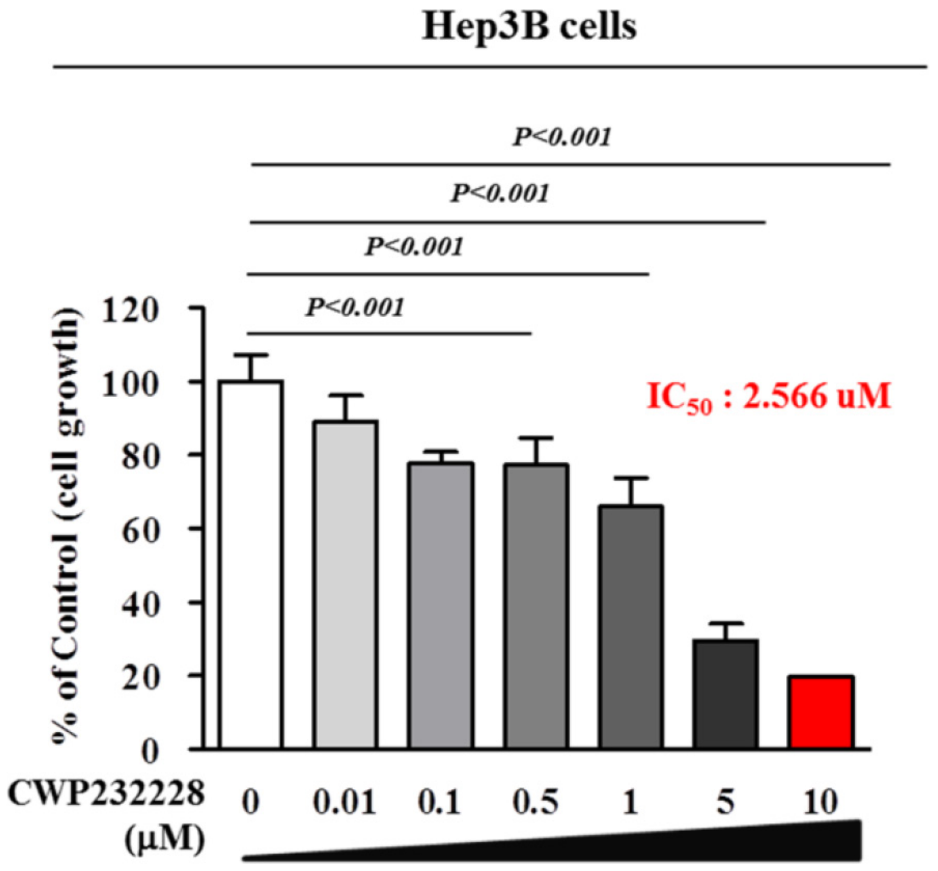

Supplementary Figure S5: IC_{50}, the concentration that inhibits 50% of proliferation in Hep3B cells. Inhibition of cell viability by CWP232228 treatment for 48 hours was determined by a CCK-8 assay (mitochondrial dehydrogenase activity) in Hep3B cells. Cell viability (%) was calculated as a percent of the vehicle control. The results are presented as the mean ± SD from three independent experiments.

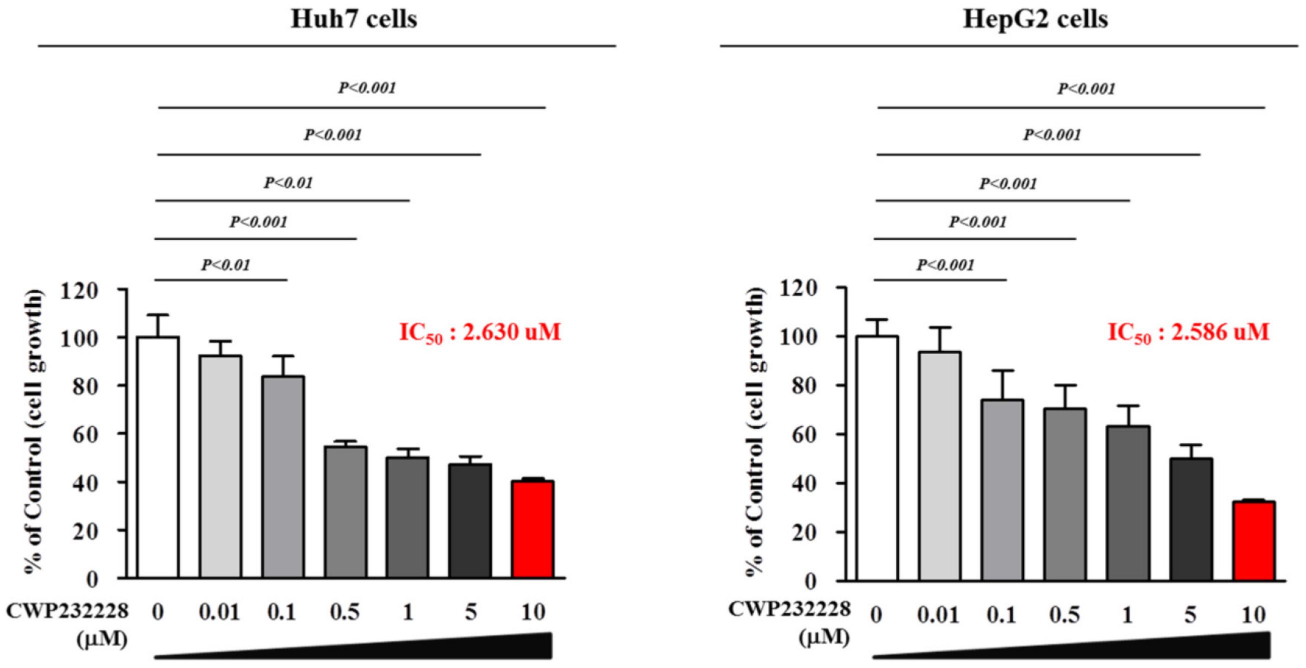

Supplementary Figure S6: IC_{50}, the concentration that inhibits 50% of proliferation in Huh7 and HepG2 cells. Inhibition of cell viability by treatment with CWP232228 for 48 hours was determined by a CCK-8 assay (mitochondrial dehydrogenase activity) in Huh7 and HepG2 cells. Cell viability (%) was calculated as a percent of the vehicle control. The results are presented as the mean ± SD from three independent experiments.

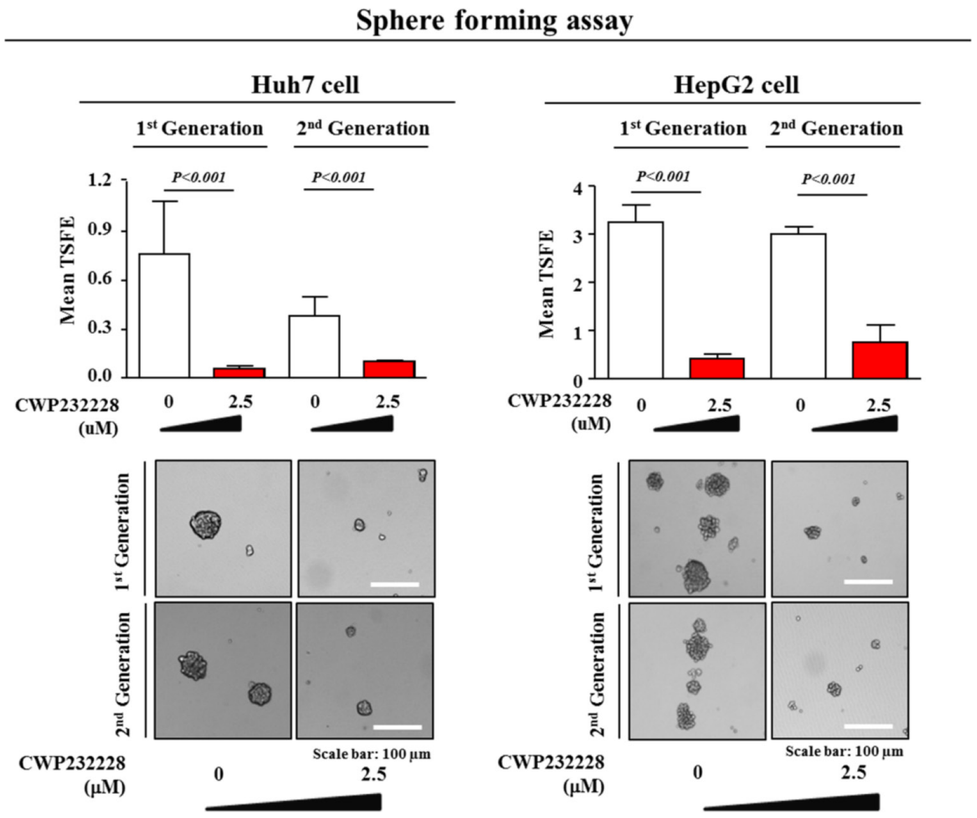

Supplementary Figure S7: Effects of CWP232228 on the sphere-forming ability in Huh7 and HepG2 cells. CWP232228 significantly inhibited primary (with CWP232228 treatment) and secondary sphere formation (without additional CWP232228 treatment) in Huh7 and HepG2 cells. The tumor sphere sizes greater than 100 μm were enumerated, and a representative image of a tumor sphere is shown. The data represent the means ± SD from three independent experiments.

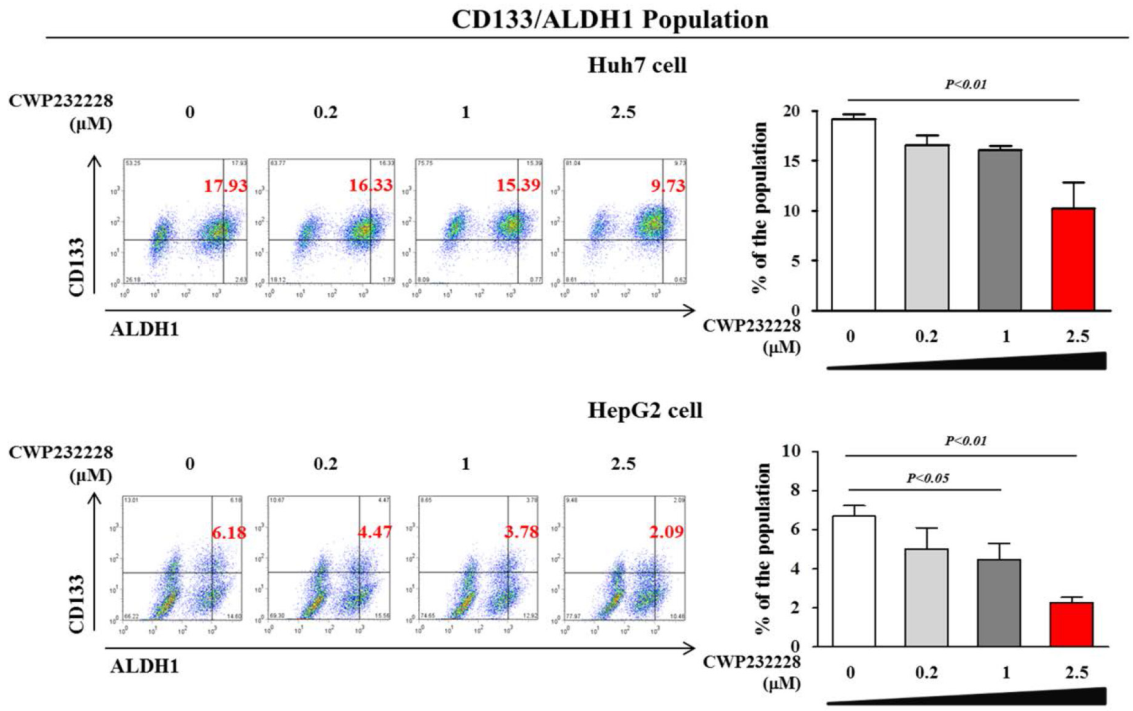

Supplementary Figure S8: Effects of CWP232228 on the expression patterns of stemness-related genes in Huh7 and HepG2 cells. Both Huh7 and HepG2 cells were treated with CWP232228 for 48 hours. The percentage of ALDH1 and CD133 subpopulations were evaluated by FACS analysis. The percentage of ALDH1 and CD133-double positive cells are significantly decreased in a dose-dependent manner. The data represent the means ± SD from three independent experiments.

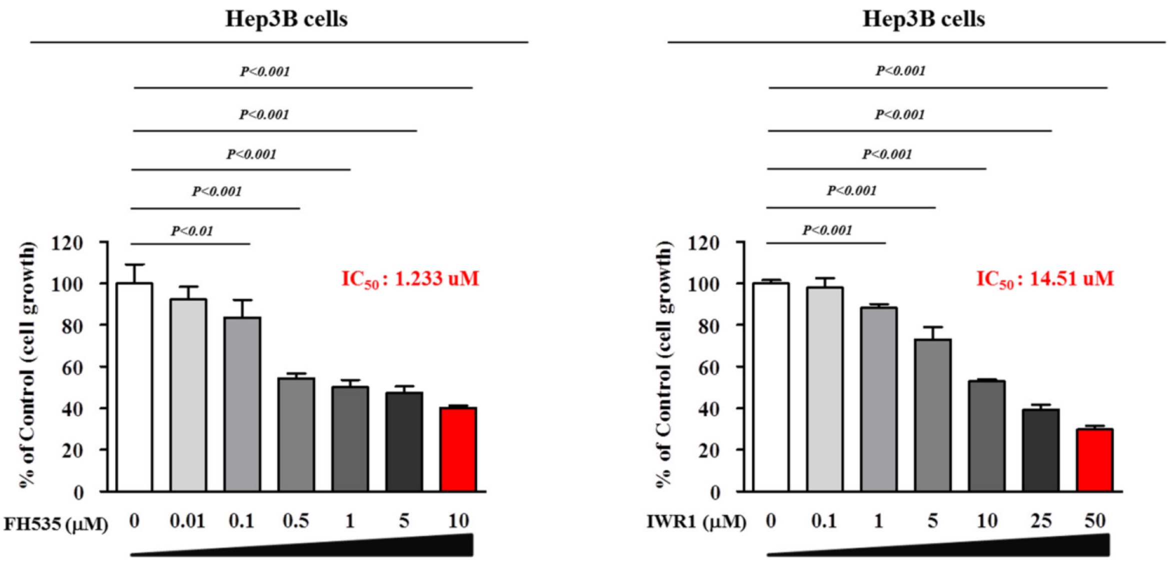

Supplementary Figure S9: IC_{50}, the concentration that inhibits 50% of proliferation in Hep3B cells. Inhibition of cell viability by treatment with FH535 and IWR1 for 48 hours was determined by a CCK-8 assay (mitochondrial dehydrogenase activity) in Hep3B cells. Cell viability (%) was calculated as a percent of the vehicle control. The data represent the means ± SD from three independent experiments.

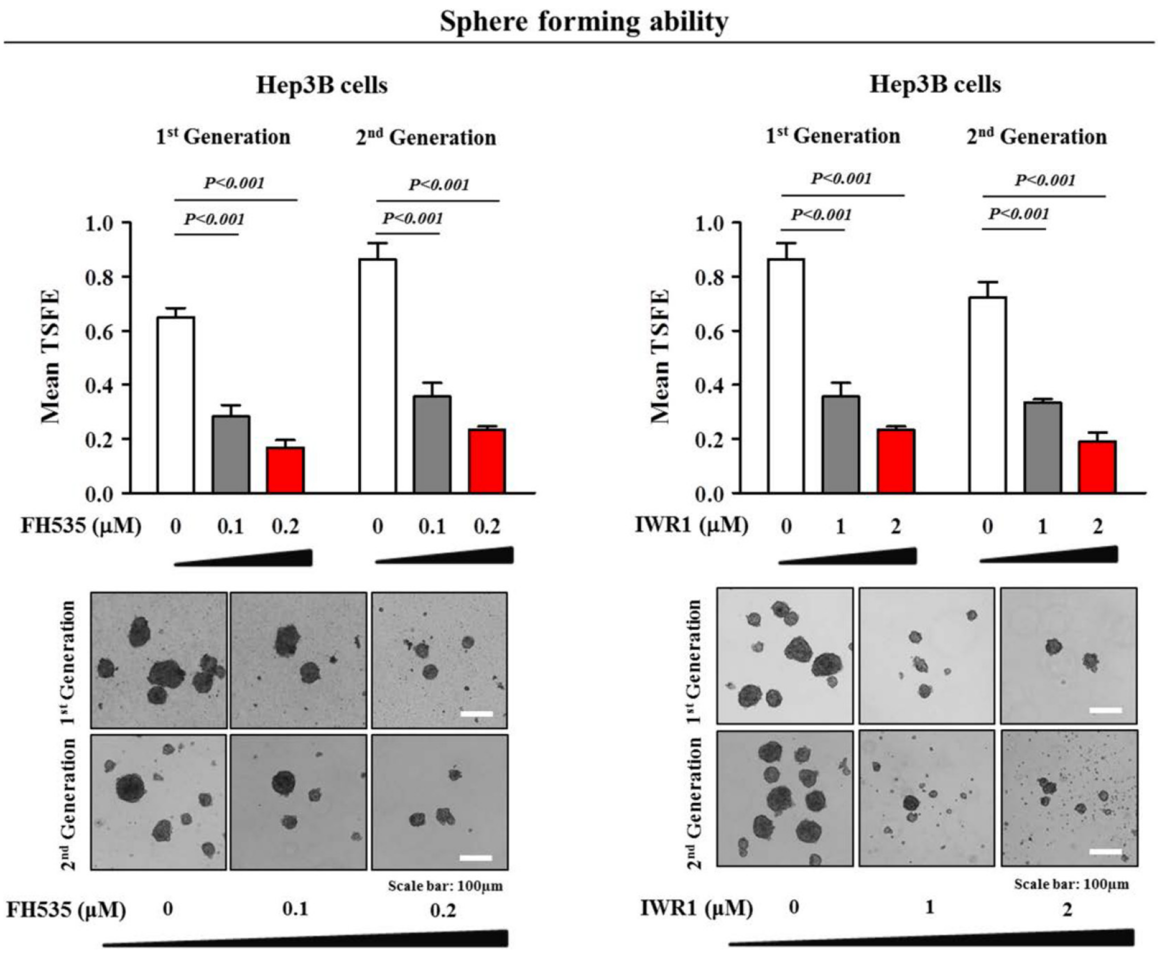

Supplementary Figure S10: Effects of FH535 or IWR1 on the sphere-forming ability in Hep3B cells. FH535 or IWR1 treatment significantly inhibited primary (with FH535 or IWR1 treatment) and secondary sphere formation (without additional FH535 or IWR1 treatment) in Hep3B cells. The tumor sphere sizes greater than 100 μm were enumerated, and a representative image of a tumor sphere is shown. The data represent the means ± SD from three independent experiments.

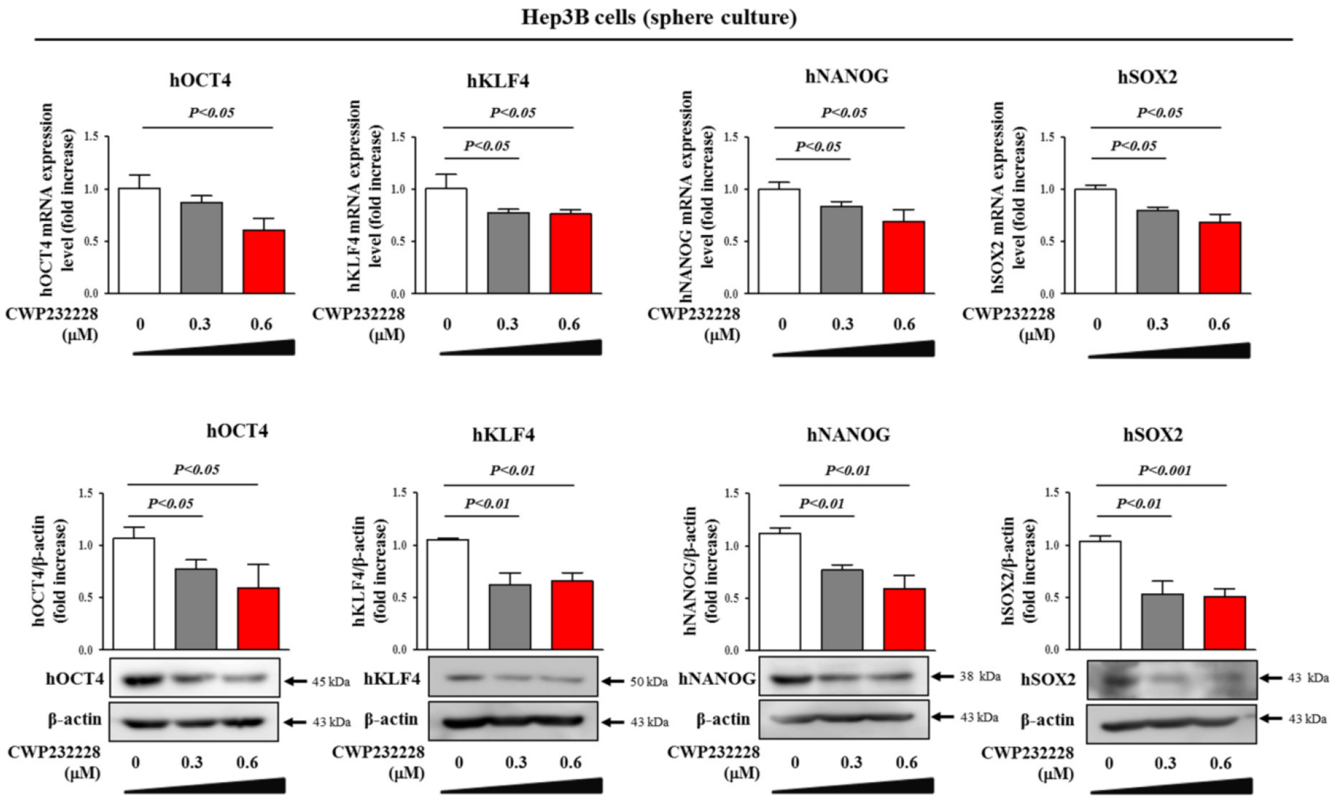

Supplementary Figure S11: Effects of CWP232228 on the expression profiles of stemness-related genes in sphere forming Hep3B cell subpopulations. After CWP232227 treatment, the mRNA and protein levels of SOX2, OCT4, KLF4, and NANOG in sphere forming Hep3B cell were measured using real-time PCR and western blotting. The mRNA and protein levels of these stemness-related genes are significantly decreased in a dose-dependent manner. The data represent the means ± SD from three independent experiments.
